# Renal shear wave elastography and urinary procollagen type III amino-terminal propeptide (uPIIINP) in feline chronic kidney disease

**DOI:** 10.1186/s12917-019-1801-4

**Published:** 2019-02-11

**Authors:** Chutimon Thanaboonnipat, Saikaew Sutayatram, Chollada Buranakarl, Nan Choisunirachon

**Affiliations:** 1 Department of Veterinary Physiology, Faculty of Veterinary Science, Chulalongkorn University, Bangkok, Thailand; 2 Department of Veterinary Surgery, Faculty of Veterinary Science, Chulalongkorn University, 39 Henri-Dunant Road, Wangmai, Pathumwan, Bangkok, 10330 Thailand

**Keywords:** Cat, Chronic kidney disease (CKD), Urinary procollagen type III amino-terminal propeptide (uPIIINP), Renal fibrosis, Shear wave elastography (SWE)

## Abstract

**Background:**

Chronic kidney disease (CKD) is one of the most common diseases occurring in cats. It is characterized by renal fibrosis, which is strongly correlated with impairment of renal function. Since renal biopsy is not performed routinely in clinical practice, the non-invasive method of ultrasonographic shear-wave elastography (SWE) was used to determine renal parenchymal stiffness. Currently, urinary procollagen type III amino-terminal propeptide (uPIIINP) is a renal fibrosis biomarker in humans. Moreover, PIIINP is increasingly applied for identification of fibrosis in various organs in animals.

**Results:**

The Young’s modulus (E) value on SWE, uPIIINP, and renal function were evaluated in 23 CKD cats and 25 healthy cats (HC). The renal cortical E values were significantly higher than those of the renal medulla in both groups (*P* < 0.001). The E values of the renal cortex and medulla were significantly higher in CKD cats than in HC (*P* < 0.001 and *P* < 0.01, respectively). The E values, especially of the cortex, showed a significant positive correlation with concentrations of plasma creatinine (*P* < 0.001), blood urea nitrogen (*P* < 0.05), while they had a negative correlation with urine specific gravity (*P* < 0.001) and urine osmolality per plasma osmolality ratio (*P* < 0.01). The uPIIINP to creatinine ratios (uPIIINP/Cr) were significantly higher in CKD cats than in HC (*P* < 0.01) and were highly correlated with renal cortical E values (*P* < 0.001).

**Conclusions:**

SWE might be an additively useful and non-invasive diagnostic imaging tool to evaluate renal parenchymal stiffness, which correlates with renal functional impairment in CKD cats. Moreover, the uPIIINP/Cr might be a promissing biomarker for adjunctive assessing the renal fibrosis in feline CKD.

## Background

Chronic kidney disease (CKD) is a common disease in cats and has been increasingly diagnosed annually, particularly in aging cats [1–5]. Renal fibrosis is the hallmark of advanced CKD and strongly correlates with renal function impairment in humans and cats [6, 7]. Renal fibrosis causes an irreversible loss of normal tissue, diminished renal function, and end-stage renal disease (ESRD). Renal biopsy is the gold standard for diagnosing renal fibrosis [8]. However, it requires anesthesia and can cause several complications. The most common complication of renal biopsy is hemorrhage. Therefore, it is not performed routinely in clinical practice particularly in high-risk patients such as CKD cats [9].

Cats with CKD normally display non-specific signs [10, 11], and routine laboratory tests, such as blood creatinine concentration, are not sensitive enough for early CKD detection [12]. Abdominal ultrasonography can provide information on the renal parenchyma that is important for identifying the underlying causes of CKD. However, typical ultrasonographic findings, such as small and irregular kidneys or increased echogenicity of the cortex and/or medulla [13–16], are not specific for CKD, and are sometimes found in healthy cats [17].

Several methods are currently used in human medicine for the determination of renal fibrosis. One of the renal fibrosis biomarkers is urinary procollagen type III amino-terminal propeptide (uPIIINP) [8, 18]. Previous studies in people have reported that high urine PIIINP to creatinine ratio (uPIIINP/Cr) was highly associated with the severity of renal fibrosis and CKD progression [8, 19, 20]. Moreover, in animals, PIIINP was also used as a fibrosis marker for assessing cardiac remodeling [21], idiopathic pulmonary fibrosis [22], and liver fibrosis in dogs [23].

In addition, renal ultrasonographic elastography (USE) is an imaging technique that has been increasingly applied in human medicine [24, 25]. USE can evaluate tissue elasticity by differentiating between normal tissue and stiff tissue, which cannot be done using hyperechogenic images from B-mode ultrasonography [26]. USE examines the tissue hardness [27, 28], using tissue elasticity evaluation that is inversely proportional to stiffness [29]. As previously described, fibrous tissue infiltration in the renal parenchyma results in decreased elasticity [30]. The two main techniques of USE available are strain elastography (SE) and shear wave elastography (SWE) [26]. In human medicine, SWE has been widely performed to assess the renal parenchymal stiffness through the Young’s modulus (E) in CKD patients [25, 31]. Several studies have reported that human patients with CKD had renal E values significantly higher than those of healthy individuals [25, 31]. Therefore, renal USE shows promise as a diagnostic imaging method to detect the early stage of CKD [28, 32, 33].

In veterinary medicine, especially in cats, the use of uPIIINP has not been reported, and reports on the use of USE are limited and only include the use of SE on normal kidneys [34]. Therefore, the purposes of this study were, first, to compare the renal tissue stiffness observed through E values between healthy cats and CKD cats using SWE; second to evaluate the relationships between the renal tissue stiffness and functional renal parameters, including plasma creatinine concentration, blood urea nitrogen (BUN), urine specific gravity (USG), urine protein creatinine (UPC) ratio, urine osmolality to plasma osmolality (Uosm/Posm) ratio, and fractional excretion of sodium, potassium, chloride, and magnesium (FE_Na_, FE_K_, FE_Cl_, and FE_Mg_) in both groups; and third to determine the relationship between uPIIINP/Cr levels and blood pressure, UPC ratio or renal E values.

## Methods

### General materials

This clinical, cross-sectional study was approved by the Chulalongkorn University Animal Care and Use Committee (CU-ACUC), Faculty of Veterinary Science, Chulalongkorn University (Protocol number: 1731055). All client-owned cats, including healthy cats (HC) and CKD cats that presented to the Small Animal Teaching Hospital, Faculty of Veterinary Science, Chulalongkorn University from September to December 2017, were considered for inclusion. The cats were considered to be HC if the history, physical examination, routine blood examinations, including complete blood count (CBC) and plasma biochemistry, USG, urinalysis, UPC ratio, abdominal radiographs, and abdominal ultrasound were normal. All cats tested negative for Feline Immunodeficiency Virus (FIV) and Feline Leukemia Virus (FeLV) infection using ELISA Test Kits (WITNESS® FeLV-FIV, Zoetis, New Jersey, USA). The CKD cats included in this study were defined based on a history of either structural or functional abnormalities of the kidneys for more than 3 months (stable CKD patients) and were categorized into IRIS stage 2, 3 and 4 CKD according to the International Renal Interest Society (IRIS) [35] staging system, in which the plasma creatinine concentrations equal to or higher than 1.6 mg/dl. Additionally, all CKD cats underwent the same diagnostic procedures as the HC and had negative results for FIV and FeLV infection from ELISA Test Kits (WITNESS® FeLV-FIV, Zoetis, New Jersey, USA). Cats with ascites, infectious diseases, lymphoma, congenital kidney diseases, such as polycystic kidney disease, hydronephrosis, generalized peripheral edema, or nephroliths were excluded from this study.

A total of 3 ml of blood was collected from each cat and placed in an EDTA tube for complete blood count (CBC) and a heparinized tube for measurement of creatinine concentration, BUN, alanine aminotransferase (ALT), alkaline phosphatase (ALP), total protein, albumin, osmolality, and electrolytes (Na^+^, K^−^, Cl^−^ and Mg^2+^). Urine was collected by voiding or catheterization for measuring the concentrations of creatinine, protein, electrolytes (Na^+^, K^−^, Cl^−^ and Mg^2+^), PIIINP, and osmolality. Indirect blood pressure in all cats was measured three times at the proximal hind leg or distal forelimb and the results were averaged. All ultrasonographic procedures were performed within a visit day.

### Analytical procedures

Concentrations of creatinine, BUN, ALT, ALP, total protein, and albumin were determined within an hour of collection by automated analyzer (The IL ILab 650 Chemistry Analyzer, Diamond Diagnostic, MA, USA). Frozen plasma and frozen urine samples (− 20 °C) were thawed to measure the osmolality and electrolytes concentration within a few weeks of collection. The plasma and urine osmolality were measured using automatic cryoscopic osmometer (OSMOMAT® 030, Gonotec GmbH, Berlin, Germany), the plasma concentrations of electrolytes were analyzed using automated analyzer (ARCHITECT c16000 clinical chemistry analyzer, Abbott Laboratories, IL, USA) and the urine concentrations of electrolytes were analyzed using automated analyzer (COBAS INTEGRA® 400 plus, Roche Diagnostics, Rotkreuz, Switzerland). USG was determined within an hour of collection using a refractometer (Master Refractometer; ATAGO®, Tokyo, Japan). Measurement of blood pressure was performed using a Doppler blood pressure instrument (Vet-Dop 2™, Vmed Technology, WA, USA). The microscopic observation of the urine sediment was performed prior to the measurement of the UPC to exclude the urinary tract infection. The UPC ratio was performed by measuring urine protein concentrations after precipitation with 3% sulphosalicylic acid [36]. Fractional excretions (FE_Na_, FE_K_, FE_Cl_, and FE_Mg_) were calculated using the formula as previously described [36]. Urinary PIIINP concentrations were determined in frozen urine samples (− 80 °C) within 2 months of collection using a sandwich enzyme-linked immunosorbent assay (ELISA) kit (Cat Procollagen Type III N-Terminal Propeptide (PIIINP) ELISA Kit, Cat. No. MBS060292, MyBioSource®, CA, USA), following the manufacturer’s instructions. Urine samples were assayed in duplicate, with an intra-assay and inter-assay coefficient of variation less than 15%. The uPIIINP/Cr levels were calculated and expressed as ng/mgCr.

### SWE procedure

The ultrasonographic examinations were performed without sedation. The cats were manually restrained and positioned in right and left lateral recumbency for each ipsilateral site of kidneys. Routine preparation for ultrasonographic examination, including hair clipping and acoustic gel coupling, was performed. All cats were screened with B-mode abdominal ultrasound, and the sagittal, dorsal, and transverse planes of the kidneys were recorded using a 9 MHz-bandwidth of a linear transducer (Resona-7, Mindray Medical International, Shenzhen, China). SWE was then performed on the sagittal plane of each kidney. The elastograms were displayed using a dual-screen, consisting of a B-mode image and an SWE color quantitative elastogram overlaid on B-mode. The fixed region of interest (ROI) was adjusted as wide as possible to cover both the renal cortex and renal medulla while attempting to control the high reliability (RLB) map during the procedure. The RLB map indicates the quality of shear wave image in a number percentage. The elasticity results measured from shear wave images with higher RLB percentages are more reliable. The elastogram color scale expressed a red color to represent the hardest tissues, whereas a blue color represented the softest tissues.

**Fig. 1:**
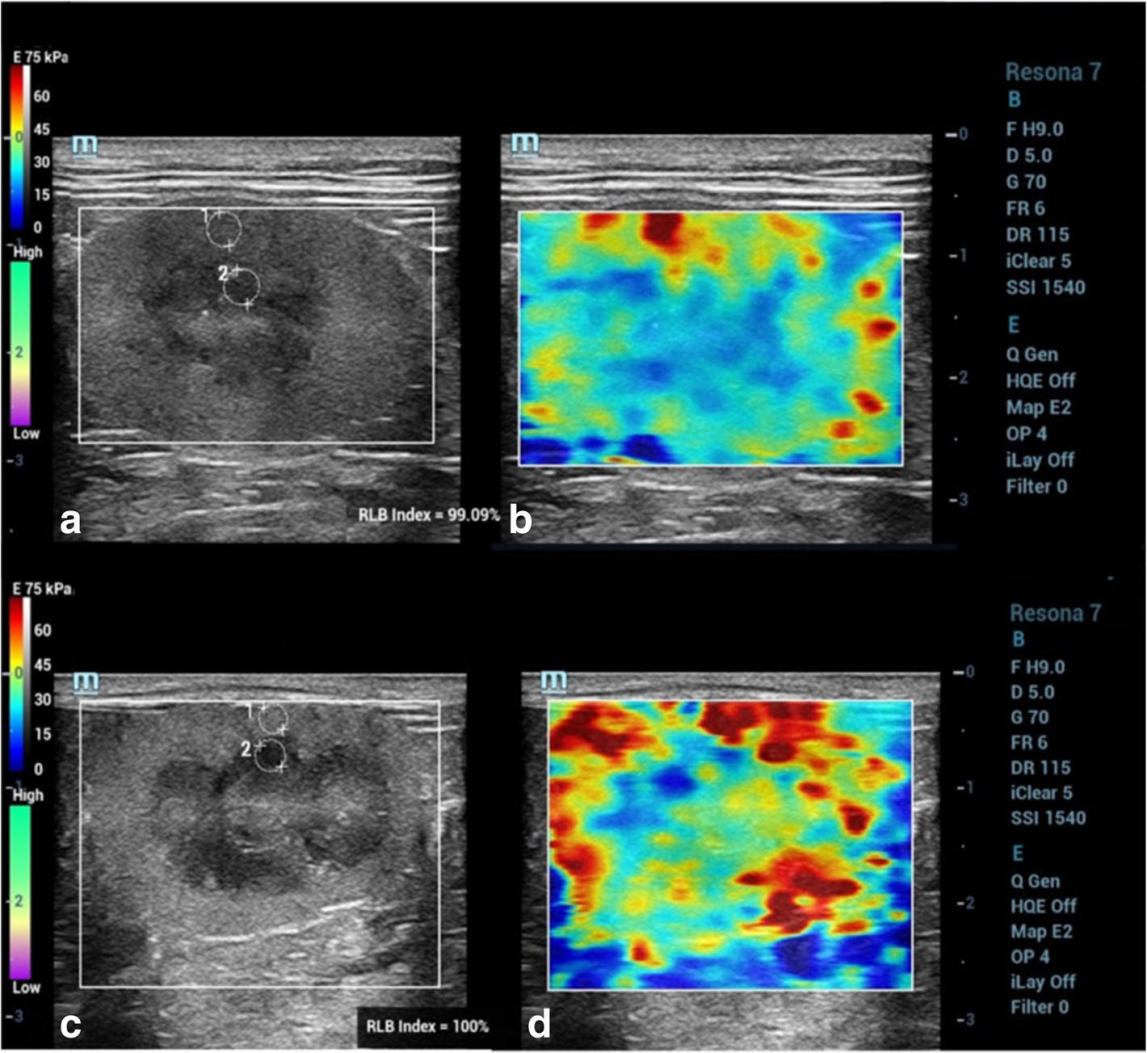
B-mode ultrasound images; **a**, **c** and color quantitative elastogram overlaid on the ROI on the B-mode; **b**, **d** between a healthy cat (HC); **a**, **b** comparing to a chronic kidney disease (CKD) cat; **c**, **d**. Regions of interest (ROIs) were drawn over the mid renal cortex and medulla to measure the Young’s modulus (E) value

The E value in kilopascal (kPa) unit was selected for representation of the tissue stiffness [37]. A stiff material has a higher E value than a soft material [37, 38]. Three separate elastograms were selected, and E values were measured at the superficial, mid renal parenchymal region using a circular electrical caliper which circular size varied according to the thickness of the renal cortical and medullary parenchyma in both HC and CKD cats (Fig. 1), and the measurements from each elastogram were averaged.

### Statistical analysis

Statistical analyses were performed by GraphPad Prism 7 software (GraphPad Software; CA; USA). Results were expressed as mean ± standard deviation (SD). Normality distributions were tested with the Shapiro–Wilk test. The average E value results between left and right kidneys in each group were analyzed by paired t-test. The average E values and uPIIINP/Cr levels were compared between groups by the unpaired t-test or Mann Whitney test. Pearson’s correlation coefficient and Spearman’s correlation coefficient were used to investigate the correlations between parameters. Statistical significance was considered if the *P*-value was less than 0.05.

## Results

A total of 25 HC and 23 CKD cats were included in this study. The mean ages of the HC and CKD cats were 4.5 ± 3.4 years (median 3 years; range 8 months–10 years) and 9.7 ± 5.1 years (median 9 years; range 3–21 years), respectively. There were 11 females (4 intact and 7 neutered) and 14 males (6 intact and 8 neutered) in the HC group, and there were 15 females (10 intact and 5 neutered) and 8 males (2 intact and 6 neutered) in the CKD group. The mean bodyweight of HC was 4.1 ± 0.9 kg (range 2.9–5.9 kg), while the mean body weight of CKD cats was 3.7 ± 0.9 kg (range; 2.6–5.9). The body condition scores (BCS) of both groups were comparable and ranged from a score of 2–4 out of 5 in each group, with a mean of 3.2 ± 0.6 and 3.0 ± 0.4 for HC and CKD cats, respectively. The HC consisted of Domestic shorthair (18), Persian (5), and American shorthair (2), and the CKD cats were Domestic shorthair (22) and Persian (1). The CKD cats were belonging to IRIS stage 2 (82.6%), stage 3 (13.1%) and stage 4 (4.3%).

Ultrasonographically, the kidneys in HC had normal shape, contour, echogenicity, and echotexture, with a mean length of 3.6 ± 0.4 and 3.5 ± 0.4 cm for the right and left kidneys, respectively. In contrast, CKD cats had irregular renal outlines, increased echogenicity of the cortex and/or medulla, and decreased corticomedullary demarcation. The CKD cats also had significantly shorter renal length (mean length of 3.1 ± 0.5 and 3.1 ± 0.6 cm for the right and left kidneys, respectively) than the HC (Right kidney; *P* < 0.01 and Left kidney; *P* < 0.001).

The average blood pressure of HC group was significantly lower than that of CKD cats (129.2 ± 17.3 mmHg and 150.9 ± 26.4 mmHg, respectively; *P* < 0.01). Urine collection was achieved in 39 cats, which were 19 HC, and 20 CKD cats. The functional renal parameters of both groups, including plasma creatinine concentration, BUN, USG, UPC ratio, Uosm/Posm ratio, FE_Na_, FE_K_, FE_Cl_, and FE_Mg,_ are summarized in Table 1. Plasma creatinine, BUN concentration and UPC ratio of CKD cats were significantly higher than those of HC (*P* < 0.001), while USG and Uosm/Posm ratio of CKD cats were significantly lower than those of HC (*P* < 0.001). All FE of electrolytes, except FE_Na_, were significantly higher in CKD cats than in HC (*P* < 0.001).

**Table 1:** Functional renal parameters include concentrations of plasma creatinine and BUN, USG, UPC ratio, Uosm/Posm ratio and FE_Na_, FE_K_, FE_Cl_, FE_Mg_ of the healthy cats (HC) and chronic kidney disease (CKD) cats

Parameters	No. of s samples	Healthy cats	CKD cats	Reference intervals
Plasma creatinine (mg/dL)	48	1.36 ± 0.19	2.59 ± 0.99^***^	0.6–1.6
BUN (mg/dL)	48	22.39 ± 4.62	39.74 ± 22.79^***^	14–36
USG	39	1.055 ± 0.01	1.034 ± 0.015^***^	1.035–1.060
UPC ratio	9	0.09 ± 0.1	0.96 ± 1.71^***^	< 0.2^a^
Uosm/Posm ratio	39	5.95 ± 1.56	2.95 ± 2.12^***^	NA
FE_Na_ (%)	39	0.58 ± 0.29	2.57 ± 3.02	< 1
FE_K_ (%)	39	18.39 ± 6.41	44.69 ± 27.41^***^	< 20
FE_Cl_ (%)	39	1.08 ± 0.47	4.79 ± 4.56^***^	< 1
FE_Mg_ (%)	39	2.41 ± 1.57	9.11 ± 7.86^***^	< 5.4

^a^Reference of UPC ratio in cats = non-proteinuric (< 0.2), borderline (0.2–0.4), proteinuric (> 0.4)

*BUN* blood urea nitrogen, *USG* urine specific gravity, *UPC ratio* urine protein creatinine ratio, *Uosm/Posm ratio* urine osmolality per plasma osmolality ratio, *FE*_*Na*_ fractional excretion of sodium, *FE*_*K*_ fractional excretion of potassium, *FE*_*Cl*_ fractional excretion of chloride, *FE*_*Mg*_ fractional excretion of magnesium

NA denotes no available data

Data are presented as mean ± SD

^***^Statistically difference between groups was made using Unpaired t- test, *P* < 0.001

**Fig. 2:**
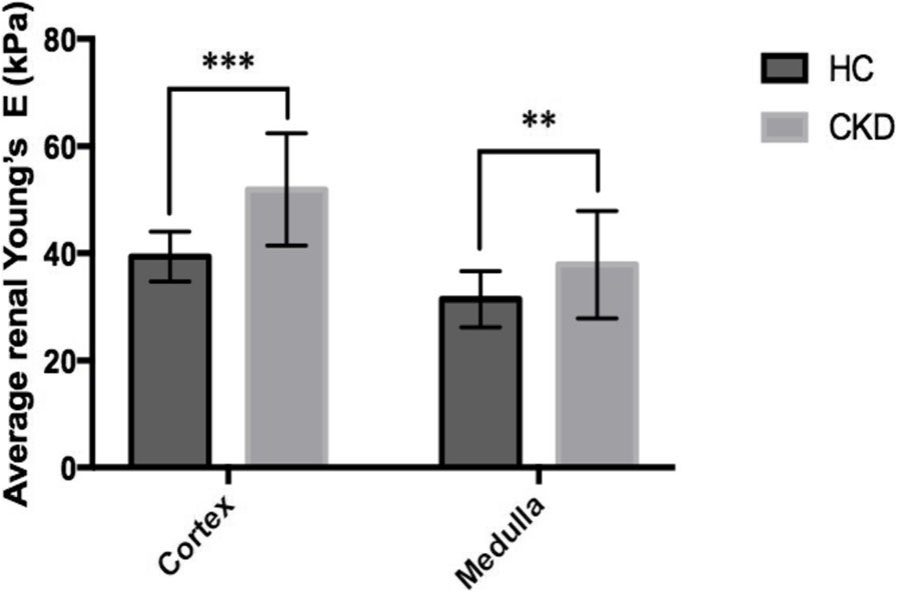
Average renal E values of cortex and medulla (mean ± SD) between the healthy cats (HC) and chronic kidney disease (CKD). *** Unpaired t-test, *P* < 0.001; ** Mann-Whitney test, *P* < 0.01. E = Young’s modulus values

The renal elasticity on the SWE of the 25 HC and 23 CKD cats were reported as average E values calculated from the right and left kidneys. The average E values of the renal cortex and medulla were not significantly different between the right and left kidneys in each group (Table 2). Age, bodyweight, and BCS did not affect the average E values of the cortex or medulla in HC. Regarding gender, the average renal E values of male cats and female cats were not significantly different. However, average renal cortical E values were significantly higher than those of the renal medulla in both groups (*P* < 0.001). Moreover, the average E values of the renal cortex and medulla of CKD cats were significantly higher than those of HC (*P <* 0.001 and *P <* 0.01, respectively) (Fig. 2).

**Table 2:** The Young’s modulus (E) values (kPa) of renal parenchyma between healthy cats group and CKD cats group

Group	E value of LK		E value of RK	
	Cortex	Medulla	Cortex	Medulla
HC	38.40 ± 7.12	30.16 ± 5.97	40.34 ± 10.42	32.73 ± 7.04
	(22.91–55.58)	(19.43–40.13)	(23.70–53.74)	(18.27–49.30)
CKD	51.89 ± 11.25	39.16 ± 13.07	51.97 ± 12.65	36.60 ± 10.06
	(22.55–74.42)	(20.19–82.13)	(23.38–75.60)	(15.38–66.51)

*E value* the Young’s modulus values in kilopascal unit, *LK* left kidney, *RK* right kidney, *HC* healthy cats group, *CKD* chronic kidney disease cats group

Data are expressed as mean ± SD and range

The average E values between left and right kidneys in each group were analyzed by Paired t-test

**Fig. 3:**
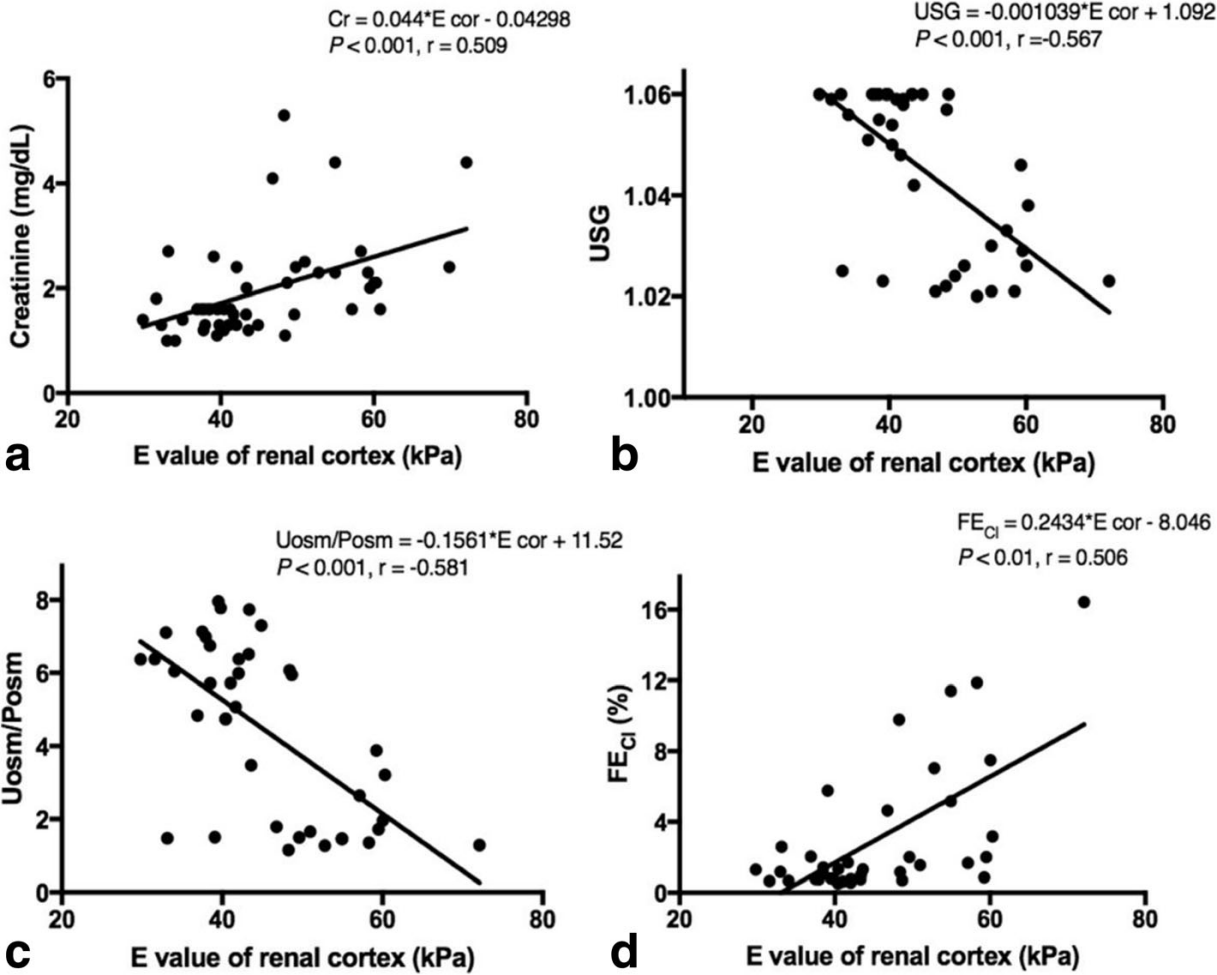
Correlations between renal cortical E values and other renal parameters using Spearman correlation; **a** average renal cortical E values and plasma creatinine; **b** average renal cortical E values and USG; **c** average renal cortical E values and Uosm/Posm; **d** average renal cortical E values and FE_Cl._ E = Young’s modulus values; USG = Urine specific gravity; Uosm/Posm = Urine osmolality per plasma osmolality; FE_Cl_ = Fractional excretion of chloride

The correlations between average renal E values and plasma creatinine concentrations or BUN in the 48 cats from both groups, and the correlations between the average renal E value and USG, UPC ratio, Uosm/Posm ratio, FE_Na_, FE_K_, FE_Cl_, or FE_Mg_ in 39 cats, from both the HC and CKD groups, were evaluated (Table 3). The E values of the cortex showed a significant positive correlation with concentrations of plasma creatinine (*P <* 0.001), BUN (*P <* 0.05), and FE_Cl_ (*P* < 0.01), while it showed a negative correlation with USG (*P <* 0.001) and Uosm/Posm (*P* < 0.01) (Fig. 3).

**Table 3:** Correlations between the average renal Young’s modulus (E) values and plasma creatinine concentration, BUN, USG, UPC ratio, Uosm/Posm ratio and FE_Na_, FE_K_, FE_Cl_, FE_Mg_ in the healthy cats (HC) and chronic kidney disease (CKD) cats

Parameters	No. of samples	Average E values of cortex	Average E values of medulla
		r	r
Plasma creatinine	48	0.509^***^	0.329^*^
BUN	48	0.361^*^	0.188
USG	39	−0.567^***^	−0.255
UPC ratio	39	0.233	−0.045
Uosm/Posm ratio	39	−0.582^***^	−0.306
FE_Na_	39	0.256	0.089
FE_K_	39	0.250	−0.219
FE_Cl_	39	0.506^**^	0.478^**^
FE_Mg_	39	0.293	0.079

*E value* the Young’s modulus values, *BUN* blood urea nitrogen, *USG* urine specific gravity, *UPC ratio* urine protein creatinine ratio, *Uosm/Posm ratio* urine osmolality plasma osmolality ratio, *FE*_*Na*_ fractional excretion of sodium, *FE*_*K*_ fractional excretion of potassium, *FE*_*Cl*_ fractional excretion of chloride, *FE*_*Mg*_ fractional excretion of magnesium

Correlations between parameters were made using Spearman correlation,^*^P < 0.05; ^**^P < 0.01; ^***^P < 0.001

**Fig. 4:**
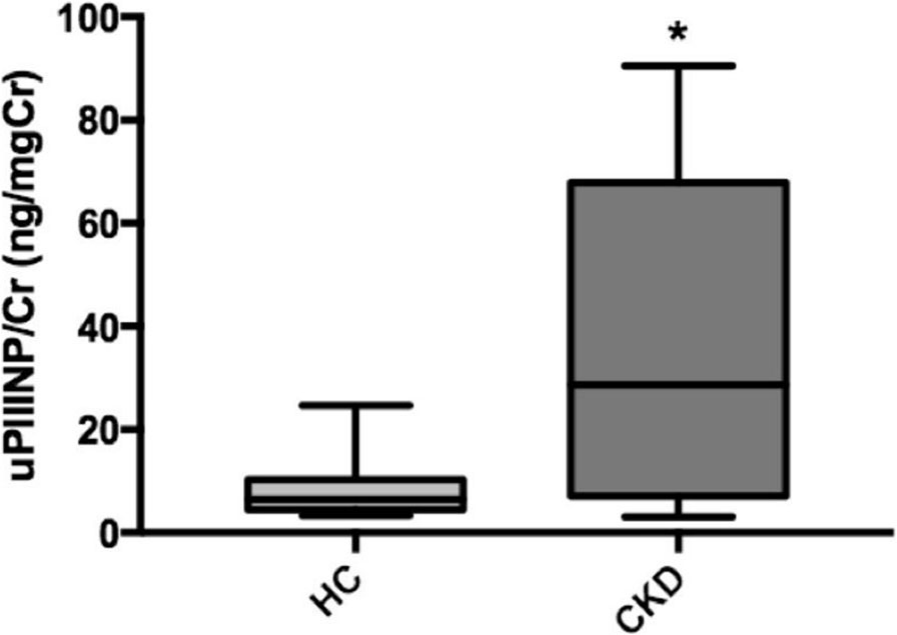
Box and whisker plot illustrating uPIIINP/Cr between the healthy cats (HC) and chronic kidney disease (CKD). * Statistically difference between groups was made using Mann-Whitney test, *P* < 0.01. uPIIINP/Cr = Urine Procollagen Type III N-Terminal Propeptide to creatinine ratio

**Fig. 5:**
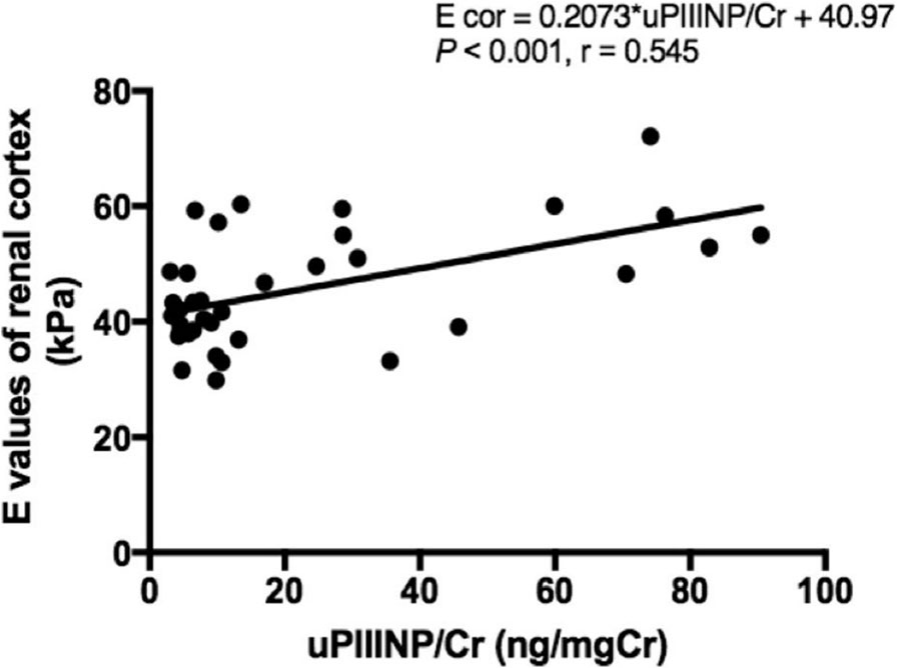
Correlation between average renal cortical E values and uPIIINP/Cr of 37 cats. Correlation was made using Pearson’s correlation coefficient. E = Young’s modulus values; uPIIINP/Cr = Urine Procollagen Type III N-Terminal Propeptide to creatinine

The average uPIIINP/Cr values of 17 HC and 20 CKD cats were compared, and the results showed that CKD cats had average uPIIINP/Cr values significantly higher than those of HC (mean uPIIINP/Cr of 34.9 ± 29.4 and 8.1 ± 5.2 ng/mgCr for CKD cats and HC, respectively; *P* < 0.01) (Fig. 4). No significant correlations were presented between uPIIINP/Cr and blood pressure or UPC ratio. A significant positive correlation (*P <* 0.001) was found between uPIIINP/Cr levels and average renal cortical E values (Fig. 5), while uPIIINP/Cr levels were not significantly correlated with average renal medullary E values.

## Discussion

USE can differentiate normal tissue from pathological tissue with an altered elasticity and increased stiffness, such as a fibrotic process [25, 39]. In veterinary medicine, the use of elastography has been described in a small number of studies on normal kidneys of various dog breeds [29] including beagles [40] and various cat breeds [34]. It has also been used to study canine mammary tumors [41, 42], and canine malignant lymph nodes [43]; however, it has not been used in renal disease.

SWE was performed in this study because it is the newest USE technique [44]; it also provides better information on elasticity than SE, in which the result is highly dependent upon operator experience [45]. Other imaging techniques have also been used to facilitate an early CKD diagnosis in cats, such as: contrast-enhanced ultrasound examination in domestic and purebred cats [46], and renal resistive index (RRI) in domestic cats [47]. However, methods such as RRI are difficult to evaluate, especially in CKD cats with renal vascular impairment and decreased renal blood flow [47]. Peng and colleagues [25] reported that human patients with CKD had stiffer kidneys than healthy people, when using SWE. Therefore, SWE may be more useful for the investigation of renal parenchymal stiffness than conventional B-mode ultrasound, especially in CKD cats.

As previously described, the renal length in cats may vary with gonadal status [48]. However, in this study, both groups contained similar numbers of intact and neutered cats. Thus, the shorter average renal length in CKD cats compared with that in HC cats was mainly due to disease progression, as decreased renal size is a common finding in advanced CKD [15].

In contrast with the results from previous studies in canine, the E values did not vary between the left and right kidneys in either group of cats. This might be because the location of the feline kidney, particularly in right kidney, interferes less with the SWE procedure compared with the location of right kidney in dogs, which is more cranial and nearer to the rib cage [40, 49]. Therefore, respiratory motion was observed to be more problematic when performing the SWE of the right kidney in comparison to the left kidney in dogs [29].

In agreement with the findings of previous studies in dogs and humans [29, 50], our results show that age, gender, bodyweight, and BCS did not affect the renal E values in HC cats. However, in this study, the small number of animals is an important limitation in evaluating the influence of demographic variables on elastography. In addition, the ages between groups were unmatched. The HC group was younger than the CKD group, because CKD has high incidence in senior cats. Therefore, the ability to obtain a HC group with a comparable age to that of the CKD group is challenging.

The elasticity of the renal cortex was significantly less than that of the renal medulla in both groups, which corresponds to SWE studies conducted in humans [25, 27] and pigs [45]. In contrast, the opposite results were reported in SE studies of healthy dogs [40] and healthy cats [34]. This disagreement among studies might result from the differences in ultrasound modalities and picture acquisition techniques. Further studies that compare between techniques would provide more clinical information of renal elasticity. From our findings, the lower elasticity of the renal cortex compared with the renal medulla could be explained by the difference in the normal physiological blood supply to the kidneys. Tissue elasticity is highly influenced by the degree of vascular pressure, and a greater proportion of the cardiac blood flow is distributed to the renal cortex than to the renal medulla [27].

Both the renal cortical and medullary E values of CKD cats were significantly higher than those of HC cats, which demonstrated that CKD cats had increased kidney stiffness. These results correspond with the studies by Guo et al. [28], Hu et al. [32], Samir et al. [33], and Peng et al. [25] in humans with CKD. However, the results of this study showed that there was an overlap between the average renal E values of HC cats and CKD cats. This might limit the use of USE for the definitive diagnosis of CKD. Rather, USE should be used as an adjunctive tool for more accurate detection and more informative monitoring of feline CKD. Furthermore, USE might assist in the diagnosis and treatment planning of renal disease in subclinical cases.

It is commonly known that renal tubulointerstitial fibrosis is the final common pathway of kidney disease [51], and is highly correlated with impairment of renal function in both humans [52, 53] and cats [54, 55]. Renal tubular functions including the absorption and excretion of water and electrolytes, were evaluated by measuring the Uosm/Posm ratio, FE_Na_, FE_K_, FE_Cl_, and FE_Mg_ in this study.

The results revealed that renal cortical E values had significant positive correlations with plasma creatinine concentrations, BUN, and FE_Cl_, while significant negative correlations existed with USG and Uosm/Posm ratio. These results correspond with the study by Lin et al. [24] in humans, in which renal elasticity was associated with a deterioration of renal function in patients with CKD. In this study, CKD cats exhibited a urinary concentrating defect leading to lower urine osmolality and Uosm/Posm ratio than in HC cats. Moreover, fractional excretion of electrolytes (FE_e_) has been reported to be a highly sensitive parameter for the evaluation of tubular impairment in dogs with advanced CKD [36]. Additionally, it has been demonstrated that FE_Mg_ may be a useful marker of the severity of tubular cell damage, as it has been positively correlated with the stage of tubulointerstitial fibrosis in humans [56].

Additionally, our findings indicate that the renal cortical E value was predominantly correlated with renal functions compared with that of the renal medullary E value. The reason for this could be that the renal cortex contains the glomeruli, which are responsible for plasma filtration [57], as well as the proximal tubules, which are responsible for the reabsorption and secretion of substances, particularly of electrolytes [58].

However, the urine osmolality and FE_e_ vary depending on several exogenous and endogenous factors especially diet and hydration status [59]. In this study, we attempted to avoid the influence of hydration status through complete history taking and physical examination, and only included normally hydrated cats in our study. However, the effect of food on these parameters could not be controlled in this study, as the use of a prescription diet is a clinical recommendation in the management of CKD. Therefore, this might be an additional limitation of our study.

The uPIIINP/Cr ratio, which was strongly correlated with the progression of interstitial fibrosis in kidney biopsies from human patients [8, 18, 19], was significantly higher in CKD cats than in HC cats. This corresponds to the results of human studies [8, 18, 19], and the results of canine studies that used PIIINP as a marker of fibrosis in cardiac remodeling [21], idiopathic pulmonary fibrosis [22], and liver fibrosis [23]. Moreover, no significant correlation was found between the uPIIINP/Cr and blood pressure or UPC ratio. This also corresponds to the results of human research, in which uPIIINP was not dependent on the degree of proteinuria [19].

Urinary transforming growth factor beta 1 (TGF-β1) is considered the most popular mediator for detecting the severity of renal fibrosis in cats [60]. However, the current gold standard method for identifying fibrosis is still a kidney biopsy [8, 61]. Although, it has been reported that urinary TGF-β1 concentration is increased in CKD cats [62, 63] and is correlated with histopathological evidence of interstitial fibrosis in cats [60], it has also been reported that urinary TGF-β1 shows no significant association with worsening renal function in both humans [61] and cats [60]. While, uPIIINP has been evaluated in human patients at various stages of CKD, the uPIIINP/Cr has been correlated with the degree of renal function impairment, as well as with the severity of renal fibrosis [8, 18–20]. In addition, uPIIINP levels correlated with urinary TGF-β1 in human studies [19]. Therefore, uPIIINP has been considered as one of the reliable markers of renal fibrosis in humans [8, 19]. Furthermore, as PIIINP has a low molecular weight (42 kDa), it is filtered by the glomeruli and reabsorbed in the proximal tubules [8, 18, 19]. Thus, an elevation in uPIIIINP could indicate reduced tubular reabsorption capacity due to the progression of renal fibrosis. Moreover, it has previously been shown in human studies [19] and in our study that the level of uPIIINP is not related to the degree of proteinuria. It might be of use as an adjunct biomarker for the detection of renal fibrosis in cats; however, the value of uPIIINP at each stage of renal fibrosis and the concomitant histopathology in cats is currently still unknown.

In this study, a statistically positive and moderate correlation was found between the uPIIINP/Cr level and the average renal cortical E values, suggesting that the renal cortical E value might be one of the indicators to assist in the evaluation of renal fibrosis in CKD cats.

In veterinary medicine, information concerning renal function parameters and renal elasticity in cats has not been reported. Moreover, the relationship between the uPIIINP/Cr level and renal elasticity in CKD cats has not been determined. This is the first report relating the renal elasticity, as determined with SWE, to the uPIIINP/Cr in CKD cats. This information would be useful for clinical practitioners and would be of interest to researchers in further study.

The major limitation of this study is that we did not perform a renal histopathological examination for comparison with the renal elasticity determined by USE and the uPIIINP/Cr results, due to the invasive nature of the biopsy. Therefore, further studies should be considered. The second limitation is that the number of healthy cats included in the study was not sufficient to evaluate the influence of demographic variables on SWE. Additionally, the HC group and CKD group were not age-matched. Further study into the effect of age and BCS on the renal elasticity would provide more reliable information. The third limitation is that other uncontrollable factors of client-owned cats, such as different types of commercial foods, might influence the Uosm/Posm ratio and FE_e_ results in this study. A further limitation is that we could not collect urine samples from all cats.

## Conclusions

USE can be used an additive diagnostic imaging method for the evaluation and monitoring of feline CKD. Plasma creatinine, BUN, and FE_Cl_ had a positive correlation with renal elasticity, while USG and Uosm/Posm ratio revealed a negative correlation with renal elasticity. The renal cortex in both groups had lower elasticity than the renal medulla, and the kidneys of CKD cats were stiffer than those of HC in this study. Moreover, the renal cortical E values were significantly correlated to the uPIIINP/Cr levels. The results suggest that renal SWE might be an easy-to-use diagnostic tool, which may be applied as adjunct for the detection and monitor of feline CKD. Furthermore, the uPIIINP/Cr levels are higher in CKD cats. Therefore, the uPIIINP/Cr might also be a promising biomarker for adjunctive assessing the renal fibrosis in CKD cats. However, further studies are needed to elucidate the clinical value of USE in terms of evaluation and monitoring of feline CKD and to construct a correlation between uPIIINP/Cr and degree of renal fibrosis from histopathology in cats.

## Abbreviations

BCS: Body condition score
BUN: Blood urea nitrogen
CBC: Complete blood count
CKD: Chronic kidney disease
CU-ACUC: Chulalongkorn University Animal Care and Use Committee
E: The Young’s modulus (E) value Standard deviation
ECM: Extracellular matrix
ESRD: End-stage renal disease
FE_e_: Fractional excretion of electrolyte
FE_Na_, FE_K_, FE_Cl_, and FE_Mg_: Fractional excretion of sodium, potassium, chloride, and magnesium
HC: Healthy cats
kPa: kilopascal
mmHg: Millimeter of mercury
RLB: Reliability
ROI: Region of interest
SD: Standard deviation
SE: Strain elastography
SWE: Shear-wave elastography
TGF-β1: Transforming growth factor beta 1
Uosm/Posm: Urine osmolality to plasma osmolality ratio
UPC: Urine protein creatinine
uPIIINP: Urinary procollagen type III amino-terminal propeptide
uPIIINP/Cr: Urine PIIINP to creatinine ratio
USE: Ultrasonographic elastography
USG: Urine specific gravity
